# Three-dimensional evaluation of condylar morphology after orthodontic treatment in adult patients with Class II malocclusion by cone-beam computed tomography

**DOI:** 10.1186/s12903-023-03728-y

**Published:** 2024-01-08

**Authors:** Qiutao Shi, Zhiyuan Gu, Danping Lai, Qi Dai, Fengyang Yu

**Affiliations:** 1 Department of stomatology, Ningbo No.2 Hospital, Ningbo, 315010 China; 2 School of stomatology, Zhejiang Chinese Medical University, Hangzhou, 310053 China; 3 Department of stomatology, The Second Affiliated Hospital of Zhejiang University, Hangzhou, 310005 China; 4 Department of Radiology, Ningbo No.2 Hospital, Ningbo, 315010 China; 5 Orthodontic Center, Perfect Dental Care, Hangzhou, 310051 China

**Keywords:** Cone-beam computed tomography, Temporomandibular joint disorders, Mandibular condyle

## Abstract

**Background:**

The aim of this study was to evaluate the condylar morphological changes after orthodontic treatment in adult patients with Class II malocclusion using a Cone-beam computed tomography (CBCT).

**Methods:**

Images of twenty-eight adult patients with Class II malocclusion who have no temporomandibular symptoms were involved in this study. To analyze the post-treatment changes in condylar morphology, mimics 17.0 software was used to measure several values and reconstruct the three-dimensional condyle, including height of the condyle, area and bone mineral density of the maximum axial and sagittal section, volume and bone mineral density of the three-dimensional condyle and condylar head before and after orthodontic treatment. Using SPSS 19.0 software package Paired t-test was applied for comparison of condylar morphology analysis between pre-treatment and post-treatment.

**Results:**

Height of condylar head increase significant (*P* < .05). Bone mineral density showed a decrease in the maximum axial and sagittal section, three-dimensional condyle and condylar head (*P* < .01). Evaluation of volume revealed that volume of both condyle and condylar head decrease considerably (*P* < .05). No significant difference was detected in other values ((*P* > .05).

**Conclusion:**

Condylar volume decreased and height of condylar head have changed, so we speculated that adaptive bone remodeling of the condyle occurs.

## Introduction

The role of occlusal condition in the onset of temporomandibular disorders (TMD) has been strongly debated for many years and still is the source of controversy [1, 2]. Occlusion is often considered a major cause of TMD. Numerous etiological and therapeutic theories are based on this presumed association and have been applied to justify the use of several therapeutic approaches, such as occlusal splint [3]. Conversely, many TMD experts hold opposing views, and various kinds of dental interventions, including routine orthodontic treatment, have been reported as causes of TMD [4, 5].

Previous study reported that patients undergoing orthodontic treatment have more symptoms and signs of TMD in the time ranged [6]. Whereas, few studies investigated the mechanism underlying the relationship. The morphology of the condyle is strongly associated with joint function and temporomandibular joint disease [7–9].

In the orthodontic treatment of Class II malocclusions, remodeling of the condyle frequently occurs on the articular surfaces of the TMJ to improve the position of the mandible relative to the maxilla [10–13].

However, detail condylar morphological changes after treatment still remains unclear. Thus, remodeling pattern of condylar morphology after orthodontic treatment could provide us with evidence about relationship between orthodontic treatment and TMD.

Condyle is anatomically a challenging structure to depict, for the reason of relatively low condylar bone density, diversified and complex morphology of condyles, proximity of the discus articularis and the overshadowing glenoid fossa [14]. Several studies have indicated that CBCT provides accurate and reliable linear measurements for reconstruction and imaging of dental and maxillofacial bony structures [15, 16]. With the aid of 3D CBCT reconstructions and superimpositions, both quantitative and qualitative analyses of condylar morphological changes could be accomplished [17].

The purpose of this study was to evaluate the morphological changes of condyle after orthodontic treatment in adult patients with Class II malocclusion.

## Material and methods

### Subjects

This study was approved by the Ethical Committee of Ningbo No.2 hospital. The 28 subjects involved in this study (7 males, 21 females; age 21.6 ± 7.9 years) presented with Class II malocclusion and were referred to the Orthodontic Center at Perfect Dental Care and the Department of Orthodontics, Zhejiang Chinese Medical University between January 2015 and December 2019. Inclusion criteria were as follows: (a) class II malocclusions with ANB larger than 3 degrees, (b) complete CBCT data were available at T0 and T1. Subjects were excluded if they presented skeletal asymmetries, unilateral posterior crossbite, articular noises at clinical examination (opening–closing), capsular or muscle pain, history of the orofacial trauma, articular systemic pathology, and history of orthodontic treatment. The average duration of treatment was 25 months.

### Radiographic examination

Morphological changes of condyle were retrospectively evaluated on CBCT images that had been taken before and after orthodontic treatment.

Images of pre-treatment and post-treatment were taken in a standard orientation: the patients were sitting in an upright position, with the occlusion plane parallel to the ground. They were instructed to breathe peacefully and avoid swallowing during the scanning process.

CBCT datasets were acquired by using a CBCT scanner (Kodak 9500, Kodak Dental Systems, Carestream Health, Los Angeles, USA) with a reconstructed slice thickness of 0.5 mm. The device was operated at 15mAs and 90 kV. A single 360°degree rotation 24-second high-resolution scan was made with 18.4*20.6 cm field of view. The imaging data were exported as Digital Imaging and Communications in Medicine (DICOM) format and the DICOM files were loaded in MIMICS 17.0 (Materialise Dental, Leuven, Belgium).

### Segmentation

**Fig. 1:**
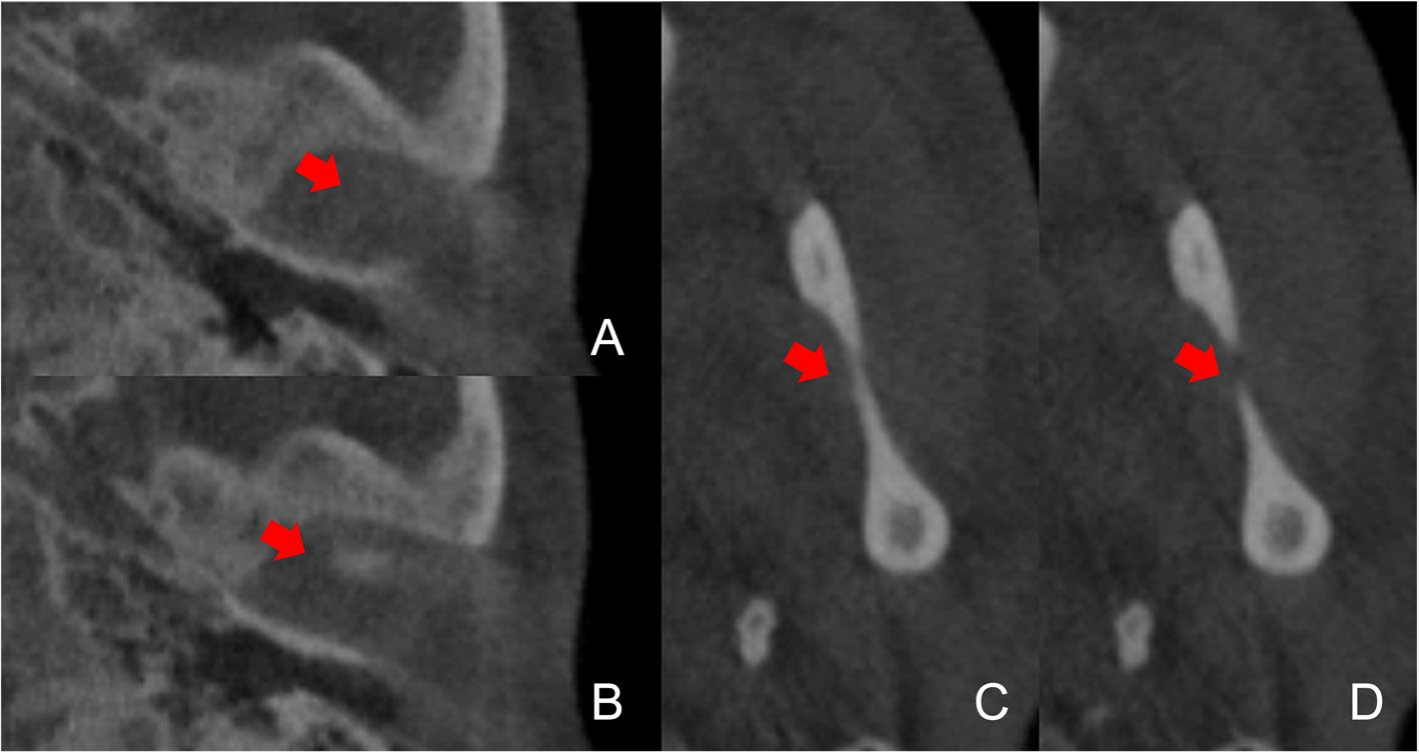
The superior and inferior limit of condyle. **A**, **B** The superior limit of condyle is selected when the first white area appears in the upper articular region (**B **red arrow); **C**, **D** The inferior limit is selected when the sigmoid area disappears (**D** red arrow)

**Fig. 2:**
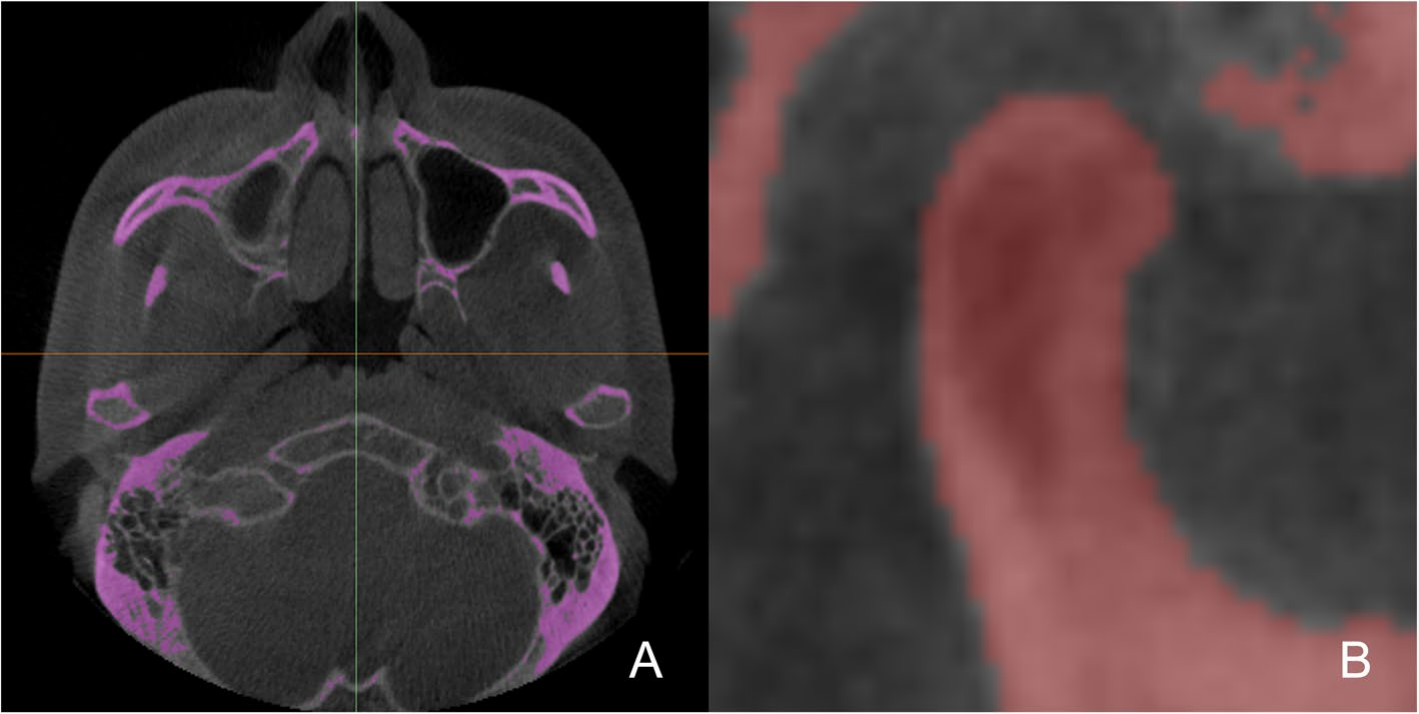
Image processing. **A** Selection of a suitable greyscale cut-off value for condyle and automatic segmentation of the condyle; **B** Manual refinement of the condylar contour on each sagittal slice

**Fig. 3:**
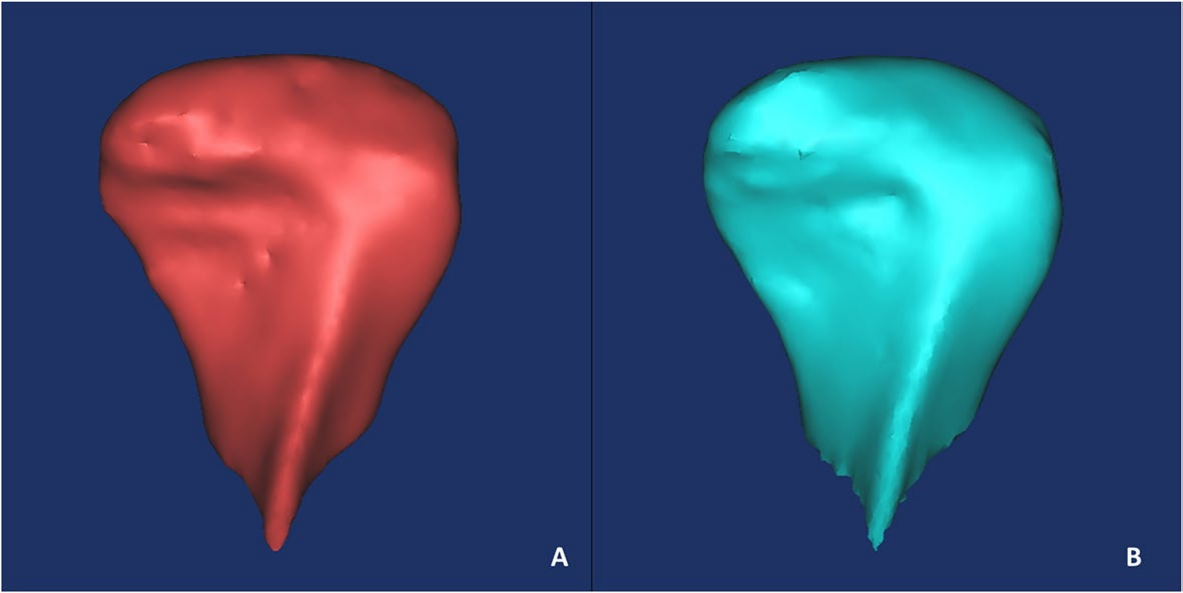
Three-dimensional reconstruction. **A** Pretreatment; **B** Posttreatment images

The superior limit of the condyle was determined where the first radiopaque point appeared in the joint space (Fig. 1A, B); The inferior limit of condyle was determined when the sigmoid notch which is between the mandibular condyle and the coronoid process disappeared (Fig. 1C, D). After determination of the conture of interest (VOI), an appropriate greyscale cut-off value was manually selected by scrolling the coronal slices of the condyle so that the condyle contour was visualized. Using this greyscale cut-off value, the contour of VOI was enhanced automatically (Fig. 2A). Then, made a fine adjustments manually by shading or erasing the under-contoured and over-contoured voxels using the function of EditMask. (Fig. 2B) [18]. After the isolation, three-dimensional reconstructions were performed for each condyle using a Mimics tool of calculating 3D from a mask (Fig. 3A, B). The height, superficial area, and volume were measured on the 3-D models, as has been described in previous studies [14, 19].

### Quantitative assessment of condylar morphology

- Condylar Height: Distance from superior point to the maximum axial section define as condylar height1 (CH1); Distance from the maximum axial section to sigmoid notch plane define as condylar height2 (CH2); Distance from superior point to sigmoid notch plane define as condylar height2 (CH3)
- Sectional Area Measurements: To evaluate the subtle changes in condylar morphology, areas of maximum axial section(Ar1) and sagittal section(Ar2) were sliced.
- Bone Mineral Density: Bone mineral density (BMD) was automatically calculated in Housefield units (HU). BMD of the maximum axial section was defined as HU1; BMD of the maximum sagittal section was defined as HU2; BMD of 3D condyle was defined as HU3; BMD of 3D condylar head was defined as HU4.
- Volume: Volume of 3D condyle was defined as volume1(V1); Volume of 3D condylar head was defined as volume2(V2).
  To investigate the surface remodeling of the condylar head, pre-treatment and post-treatment segmented images were superimposed using both point registration and global registration techniques, employing the least root mean square method [20].
  In order to assess the reproducibility and reliability of accuracy of measurements, all CBCT images data were assigned by a random number. Two independent residents in oral and maxillofacial surgery carried out this process and measurement in a blinded manner. The process and measurements were repeated after an intervening period of 4 weeks. The Bland-Altman method was used to evaluate the measurement error. The results show higher intra-observer and inter-observer correlation coefficients (ICC > 0.9).

## Statistical analysis

All testing was performed by the use of SPSS 22.0 software package (SPSS Inc., Chicago, IL, USA). Kolmogorov–Smirnov test was used to determine the distribution of continuous variables. Continuous variables were expressed as means+standard deviation. Difference in measurements of Ar2 and Hu2 were analyzed using Wilcoxon test. A paired t test was used to analyzed differences in all the rest parameters between T0 and T1. The *p* value was set at 0.05.

## Result

**Fig. 4:**
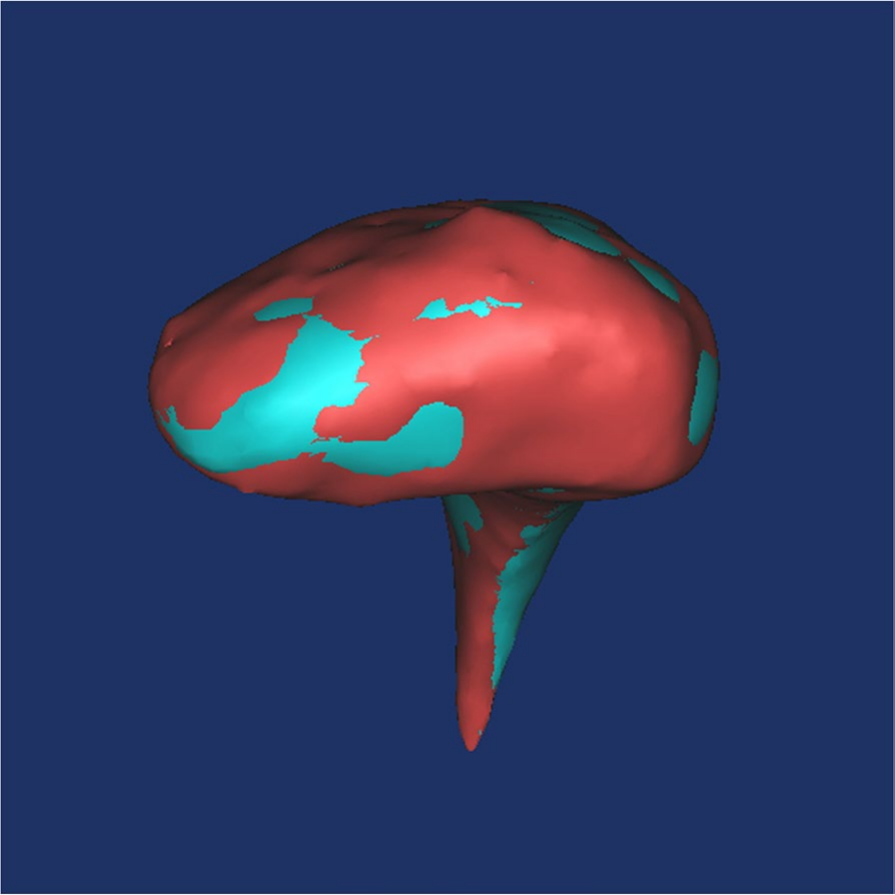
Superimposition of pre- and post-treatment models. Red region represented bone resorption; Blue region represented bone formation

Condylar head height(H1) increased significantly post-treatment (*P* < 0.05, Table 1). BMD showed a significant decrease in the maximum axil section, the maximum sagittal section, 3D condyle and condylar head (*P* < 0.01, Table 2). Evaluation of volumetric measurements revealed that 3D condyle volume (*P* < 0.01, Table 3) and condylar head volume decreased significantly post-treatment (*P* < 0.05, Table 3). No significant difference was found in other measurements (*P* > 0.05, Tables 1, 4). Superimposed pre-treatment and post-treatment images showed both bone formation and resorption on the surface of condyle (Fig. 4).

**Table 1:** Condylar height change pre- and post-treatment (mm, $\overline{x}$ ±s)

	Pre-treatment	Post-treatment	*P* value
CH1	6.72 ± 2.05	7.11 ± 1.85	0.018*
CH2	10.30 ± 2.85	10.04 ± 2.70	0.16
CH3	17.01 ± 2.91	17.15 ± 3.10	0.315

*P < 0.05 **P < 0.01

**Table 2:** The mean bone density change pre- and post- treatment (Hu, $\overline{x}$ ±s)

	Pre-treatment	Post-treatment	*P* value
Hu1	619.51 ± 123.41	550.61 ± 126.94	0.00**
Hu2^a^	623.34 ± 119.51	549.71 ± 119.90	0.00**
Hu3	794.74. ± 105.53	690.80 ± 108.29	0.00**
Hu4	626.37 ± 114.31	546.92 ± 102.55	0.00**

*P < 0.05 **P < 0.01

^a^Wilcoxon test for paired samples

**Table 3:** The volume change pre- and post-treatment (mm^3^, $\overline{x}$ ±s)

	Pre-treatment	Post-treatment	*P* value
V1	1657.66 ± 517.21	1541.49 ± 467.89	0.00**
V2	670.26 ± 316.16	618.52 ± 282.57	0.027*

*: *P* < 0.05 **P < 0.01

**Table 4:** Area of the largest axil plane change pre- and post- treatment (mm^2^, $\overline{x}$ ±s)

	Pre-treatment	Post-treatment	*P* value
Ar1	281.13 ± 55.41	276.70 ± 54.76	0.106
Ar2^a^	101.50 ± 46.33	101.93 ± 38.93	0.339

*P < 0.05 **P < 0.01

^a^Wilcoxon test for paired samples

## Discussion

In the present study, we have observed significant differences in the morphologic features of the condyle, which may offer valuable insights into condylar changes following orthodontic treatment. Condylar height is a crucial parameter for assessing condylar growth and development. In our investigation, we observed a significant increase in H1 post-treatment compared to pre-treatment, implying that the correction of distal occlusion may promote condylar growth, even in adult patients. However, differences in the area of the maximum axial and sagittal sections were not statistically significant between T0 and T1.

This study has demonstrated a significant decrease in condylar bone volume post-treatment. This indicates that condylar bone volume undergoes remodeling to adapt to changes in functional demands. Kurusu et al. [21] also noted that patients with low occlusal force tend to have smaller mandibular condyles, suggesting a correlation between occlusal force and condylar volume. In our investigation, both the volume of the 3D condyle and the condylar head decreased post-treatment. Prior studies have shown that occlusal force tends to decrease during the treatment period due to occlusal instability, with an increase expected during and after the retention phase [22, 23]. Therefore, the authors infer that the decrease in condylar bone volume may be associated with the reduction in occlusal force during treatment. As a result, condylar bone volume undergoes a remodeling process in response to changes in mechanical load. Further studies are necessary during the follow-up visits to investigate this phenomenon in more detail.

Bone mineral density (BMD) is a commonly used surrogate measure of mechanical competence and bone strength in both clinical and experimental contexts, expressed in terms of areal (aBMD) and volumetric (vBMD) measurements. In the current study, notable reductions in BMD were observed post-treatment. Chen et al. [24] discovered that a decrease in TMJ loading led to condylar cartilage loss and a transient reduction in the density of the mandibular condylar subchondral bone in mice. Furthermore, Teixeira et al.’s research revealed that dynamic loading stimulates remodeling of the mandibular condyle, potentially offering a treatment avenue for regenerating condylar cartilage and enhancing the impact of orthopedic appliances on mandibular growth [25]. It’s worth noting that the frequency of bone remodeling activation has a discernible effect on condylar BMD and its distribution [26]. Bone remodeling acts as a regulator of the degree of mineralization and the distribution of minerals at the tissue level, directly influencing the mechanical properties of bone [27]. Consequently, it can be inferred that the changes in BMD observed in our study result from the remodeling of the condyle induced by orthodontic treatment.

In our study, we utilized Hounsfield Units (HU) obtained from cone-beam computed tomography (CBCT) as a reference for bone mineral density (BMD). It is worth noting that several studies have indicated that voxel values from CBCT images may not be reliable for BMD estimation due to potential influences from the imaging device and positioning [28]. In a standard CT scan, HU is directly proportional to the degree of X-ray attenuation and is assigned to each pixel, thus representing the tissue density in the image. While CBCT manufacturers and software providers often present grayscale values as HUs, it’s important to recognize that these measurements do not correspond to true Hounsfield Units. Nevertheless, some studies have suggested a strong linear correlation between grayscale values in CBCT and HUs in conventional CT scans under controlled conditions [29, 30]. In our study, we adhered to a standardized CBCT scanning protocol both before and after treatment, which should have maintained relatively reproducible grayscale values between the scans. Therefore, any differences in grayscale values between the scans could be attributed to changes in bone density.

In the current study, the parameters, patient position, and CBCT device remained consistent between the pre-treatment and post-treatment scans. Therefore, the grayscale value, used as an indicator of BMD, can be considered relatively reliable. Nevertheless, the precise nature of its relationship warrants further examination.

For a comprehensive assessment of condylar surface changes, the utilization of 3D reconstructed images and a superimposition method is indispensable. 3D surface images provide a more precise representation of condylar remodeling compared to conventional 2D images. In the current study, the superimposition of pre-treatment and post-treatment 3D reconstructed images revealed that both bone formation and bone resorption had transpired on the condyle’s surface. This suggests an ongoing adaptation process to the altered TMJ environment.

However, due to the absence of a control group, we were unable to exclude the potential influence of age on condylar morphology. During adulthood, the condyle frequently undergoes remodeling processes that can impact its morphology [31]. Studies by Pontual et al. [32, 33] have shown an increased prevalence of degenerative bone changes with advancing age. Furthermore, it’s worth noting that 75% of the subjects in our study were female, which could introduce an additional factor affecting condylar remodeling. Epidemiological data indicates a strong female predilection for Temporomandibular Disorders (TMD), with an estimated male-to-female ratio of approximately 1:3 in TMD patients [34]. This predisposition is believed to be associated with female reproductive hormones, especially estrogen. Previous research has revealed that estrogen signaling pathways play a role in regulating pregnancy-related TMJ homeostasis [35]. A reduction in bone density can lead to TMJ degeneration. Another study found that Era polymorphism had an impact on altered mandibular dimensions in female symptomatic TMJ osteoarthritis patients, suggesting that estrogen may contribute to TMJ bone remodeling [36]. Additionally, the follow-up duration was not sufficient to capture long-term, longitudinal changes in subchondral bone in response to orthodontic treatment. Hence, future investigations should explore these long-term changes. Moreover, the study was limited by the relatively small number of patients due to its retrospective design. A well-designed prospective study can overcome this problem.

## Conclusion

In conclusion, our study demonstrates a notable decrease in condylar bone volume and bone mineral density during the course of orthodontic treatment in patients with Class II malocclusion.

## Data Availability

The datasets used and/or analyzed during the current study are available from the corresponding author on reasonable request.
